# Role of SatO2, PaO2/FiO2 Ratio and PaO2 to Predict Adverse Outcome in COVID-19: A Retrospective, Cohort Study

**DOI:** 10.3390/ijerph182111534

**Published:** 2021-11-02

**Authors:** Stefano Sartini, Laura Massobrio, Ombretta Cutuli, Paola Campodonico, Cristina Bernini, Marina Sartini, Maria Luisa Cristina, Luca Castellani, Ludovica Ceschi, Marzia Spadaro, Angelo Gratarola, Paolo Barbera

**Affiliations:** 1 Emergency Medicine Department, San Martino Policlinic University Hospital, 16132 Genoa, Italy; Stefano.sartini@hsanmartino.it (S.S.); laura.massobrio@hsanmartino.it (L.M.); Ombretta.cutuli@hsanmartino.it (O.C.); piocampo@libero.it (P.C.); Cristina.bernini@hsanmartino.it (C.B.); paolo.barbera@hsanmartino.it (P.B.); 2 Department of Health Sciences, University of Genova, 16132 Genoa, Italy; cristinaml@unige.it; 3 Hospital Hygiene, E.O. Ospedali Galliera, 16128 Genoa, Italy; 4 Emergency Medicine Post-Graduate School, University of Genoa, 16132 Genoa, Italy; luca_castellani@ymail.com (L.C.); Ludovica.ceschi91@gmail.com (L.C.); marzia.spadaro@libero.it (M.S.); 5 Division of Anesthesia and Intensive Care, San Martino Policlinic University Hospital, 16132 Genoa, Italy; angelo.gratarola@hsanmartino.it

**Keywords:** COVID-19, respiratory failure, hyperoxia, hypoxia, non-invasive ventilation

## Abstract

COVID-19 respiratory failure is a life-threatening condition. Oxygenation targets were evaluated in a non-ICU setting. In this retrospective, observational study, we enrolled all patients admitted to the University Hospital of Genoa, Italy, between 1 February and 31 May 2020 with an RT-PCR positive for SARS-CoV-2. PaO2, PaO2/FiO2 and SatO2% were collected and analyzed at time 0 and in case of admission, patients who required or not C-PAP (groups A and B) were categorized. Each measurement was correlated to adverse outcome. A total of 483 patients were enrolled, and 369 were admitted to hospital. Of these, 153 required C-PAP and 266 had an adverse outcome. Patients with PaO2 <60 and >100 had a higher rate of adverse outcome at time 0, in groups A and B (OR 2.52, 3.45, 2.01, respectively). About the PaO2/FiO2 ratio, the OR for < 300 was 3.10 at time 0, 4.01 in group A and 4.79 in group B. Similar odds were found for < 200 in any groups and < 100 except for group B (OR 11.57). SatO2 < 94% showed OR 1.34, 3.52 and 19.12 at time 0, in groups A and B, respectively. PaO2 < 60 and >100, SatO2 < 94% and PaO2/FiO2 ratio < 300 showed at least two- to three-fold correlation to adverse outcome. This may provide simple but clear targets for clinicians facing COVID-19 respiratory failure in a non ICU-setting.

## 1. Introduction

Since December 2019, SARS-CoV-2 outbreak has challenged health-care systems across the world as it is associated with high mortality and morbidity [1]. Upper respiratory tract infection, pneumonia and acute respiratory distress syndrome (ARDS) are the most common and serious causes of hospitalization and demand for critical care environment [2,3,4]. Clinically, acute hypoxemic respiratory failure is the dominant finding whilst hypercapnia is rare [5]. Facial mask oxygen, high flow nasal cannula (HFNC), helmet C-PAP (continuous positive air pressure) and non-invasive ventilation (NIV) are the alternatives to mechanical ventilation in non-intensive care unit (ICU) settings to maintain adequate level of blood oxygenation [6]. Persistent hypoxia, hyperactivation of inflammatory and immune mechanisms and hypercoagulable state are responsible for increased risk of cerebrovascular events and cardiac dysfunction [7,8]. The brain is especially sensible to oxygen content changes causing cerebral blood flow control derangements and increased anaerobic glycolysis products (lactic acid, oxygen free radicals, lipid peroxides) leading to interstitial brain edema, intracranial hypertension and decreased ATP production [9,10].

Adaptation to hypoxia and oxygen toxicity are well known mechanisms in critical ill patients, and these elements represent a challenge in the understanding and management of ARDS in COVID-19 [11,12,13]. Because of socio-cultural, organizational and personal convictions, many patients come to hospital observation after many days of symptoms onset (often more than one week) [14,15,16]. In those developing COVID-19-related respiratory failure, prolonged hypoxia causes adaptative systems activation [17]. Alveolar ventilation, cardiac output and red cell mass increase are early mechanisms to maintain tissue oxygen delivery (DO2) whilst reduction in ATP production and cellular metabolic processes downregulation represent chronic adaptative responses to cellular oxygen consumption (VO2) [18,19].

Supplemental oxygen, irrespective of the interface used, may cause a derangement of these adaptation mechanisms and direct harm to many organs through reactive oxygen species (ROS) production [20]. Moreover, supranormal arterial oxygenation has been related to adverse events like reduced myocardial function and coronary blood flow, increased mortality in stroke and septic shock and neutrophil-induced oxidative stress [21,22,23].

Peripheral oxygen saturation (SatO2) and arterial blood gas (ABG) analysis are commonly used to assess oxygenation in non-ICU settings. PaO2/FiO2 ratio is the index used to classify the severity of ARDS according to the Berlin definition even though most of the evidence derives from intensive care settings [24]. HACOR score and COX index has been proposed to predict adverse outcomes in patients on C-PAP and HFNC, respectively, but they were not considered for this study due to a lack of data and device availability [25,26]. Whilst several consensus statements were in place that recommended maintaining the lower thresholds of SatO2 ≥ 94% and PaO2 > 60 mmHg, few data were available about the “higher” threshold especially for PaO2 level in COVID-19 [27].

The primary objective of this study was to analyze SatO2, PaO2/FiO2 ratio and PaO2 values in patients with COVID-19 respiratory failure and correlate these parameters with adverse outcomes.

Secondarily, we aimed to identify SatO2, PaO2/FiO2 ratio and PaO2 relevant thresholds outcomes related to provide simple but clear targets for clinical management.

## 2. Materials and Methods

### 2.1. Study Design and Settings

This is a retrospective observational study considering all patients found having a reverse transcription of polymerase chain reaction (RT-PCR) positive for SARS-CoV-2 to a nasopharyngeal swab.

We collected data from 1 February to 31 May 2020 about all the patients admitted to the Emergency Department at the University Hospital of Genoa, Italy. This is a tertiary care hospital with full facilities and a total of 700 beds, and at the time of the study, almost 200 beds were dedicated to COVID-19 patients (of these, 25 were ICU beds, and the others were reorganized from internal medicine, infective disease, rheumatology and endocrinology wards to face the outbreak). Of each patient, informatic charts were reviewed using our unified healthcare information system (TrackCare© 1996–2021 InterSystems Corporation, Cambridge, MA, USA) from hospital admission to discharge or death. Furthermore, we looked at hospital readmission within 30 days from discharge.

The study was conducted according to the guidelines of the Declaration of Helsinki and approved by the Local Institutional Ethics Committee (CER Liguria: 460/2020—DB id 10865).

### 2.2. Particitants and Data Collection

All patients who were >18 years old with a RT-PCR positive for SARS-CoV-2 admitted to the Emergency Department were eligible for the study.

The exclusion criteria were the following: lack of data about PaO2, SatO2 and PaO2/FiO2 ratio at hospital admission; those with positive swab for SARS-CoV-2 after hospital admission; presentation not related to COVID-19 clinical feature (for example, trauma or surgical patients); direct ICU admission; expectance of survival <6 h.

For admitted patients in this peculiar pandemic situation, ICU admission criteria were: PaO2 < 60 and/or PaO2/FiO2 < 100 despite optimization of C-PAP set-up, uncontrolled respiratory distress, GCS < 9, age below life expectancy (82 years), no end-of-stage chronic disease.

C-PAP indications were: PaO2 < 60 and/or PaO2/FiO2 < 100 despite optimization of oxygen mask set-up; signs of respiratory distress.

Nobody received HFNC as this device was not available at our hospital at the time of the study.

For each patient we collected:

- Age;
- Gender;
- Coexisting disorder (hypertension, smoke, hypercholesterolemia, heart failure, COPD, pulmonary restrictive diseases, coagulopathies, immunodepression, diabetes, vascular-artery disease, chronic kidney disease, active solid cancer, active hematological disorder);
- Medications (ACE inhibitors, steroids, oral anticoagulant);
- Vital parameters at admission (systolic pressure, diastolic pressure, SatO2%, heart rate, respiratory rate, temperature);
- Laboratory test at admission (white cell count, neutrophils, lymphocytes, platelets, aPTT, INR, d-dimer, fibrinogen, CRP, procalcitonin, lactate deydrogenase, IL-6, creatine-kinase, ferritin, troponin, creatinine, NT-probnp);
- Arterial blood gas analysis: pH, pCO2, PaO2, PaO2/FiO2 ratio;
- Number of patients requiring supplemental oxygen via face mask and those requiring non-invasive ventilation/C-PAP helmet.

### 2.3. Outcomes Measures

ABG values were reviewed from admission to hospital discharge or until the finding of one of the adverse outcomes. We analyzed these data considering three different phases: first ABGs made at hospital admission in the Emergency Department (Time 0), ABGs made during hospital admission in those not on C-PAP (group A), ABGs made during hospital admission in those on C-PAP helmet (group B). Patients admitted to hospital not requiring C-PAP could have switched to C-PAP helmet or remained on face mask on the basis of clinical judgement. ABGs were made at least daily in those on face mask and more frequently for those on C-PAP helmet.

At time 0, pH, PaO2, PaO2/FiO2 and SatO2 were collected. For group A and group B, we collected the best and worst PaO2, the worst PaO2/FiO2 and SatO2 values found at any time from admission.

ABGs values were categorized as follows and matched with the presence of at least one adverse outcome:

- SatO2 < 94% versus SatO2 ≥ 94% (value chosen on the basis of WHO indication).
- PaO2/FiO2 ratio subdivided using the threshold of 100–200–300 according to the Berlin criteria of ARDS.
- PaO2 < 60 and >100 mmHG (out of normal range) versus PaO2 60–100 (in range).

As adverse outcomes we considered: invasive ventilation, ICU admission, intrahospital mortality, C-PAP-failure, hospital readmission within 30 days from discharge, length of hospital stay. We defined “C-PAP failure” the need to restart C-PAP after a weaning deemed effective.

### 2.4. Data Analysis and Statistical Methods

Patients’ characteristics were presented as median and interquartile range (IQR) for continuous variables and expressed as absolute values along with percentages for categorical variables.

Of the arterial blood gas parameters, PaO2 levels were compared and categorized in <60 mmHg, 60–100 mmHg, >100 mmHg; SatO2 in < 94 and ≥94%, PaO2/FiO2 < 100, 100–200, 200–300 and ≥300.

The population was subdivided according to the presence of at least one adverse outcome (in-hospital mortality, IOT, C-PAP failure, hospital readmission within 30 days from discharge). PaO2, SatO2 and PaO2/FiO2 categories as identified above were compared between the two sub-populations with and without adverse outcome. For the comparison we used the χ2 test or Fisher’s exact test where appropriate.

Logistic regression models were used to estimate the odds ratios (ORs) and 95% confidence intervals (CIs) to identify arterial blood gas analysis parameter independently associated with adverse outcomes. To establish the OR of PaO2 values, we assigned 0 to a PaO2 range between 60 and 100 mmHg (representing the ideal target of normoxia) and 1 to PaO2 < 60 mmHg and >100 mmHg considered values out of the favorable range. Having recalculated PaO2 continuous values into binomial variable (favorable: PaO2 60–100; non-favorable: PaO2 < 60 and >100), we calculated for each category the odds for adverse outcomes. Similarly, we calculated the OR for adverse outcomes of SatO2 <94% and of PaO2/FiO2 < 100, 100–200, 200–300 according to the international definition for the stratification of acute respiratory distress syndrome.

All tests were two-sided, and a *p* value less than 0.05 was considered statistically significant. We included all participants for whom the variables of interest were available in the final analysis, without imputing missing data. All statistical analyses were done with Stata/SE 14.2 (StataCorp, College Station, TX, USA).

## 3. Results

**Figure 1:**
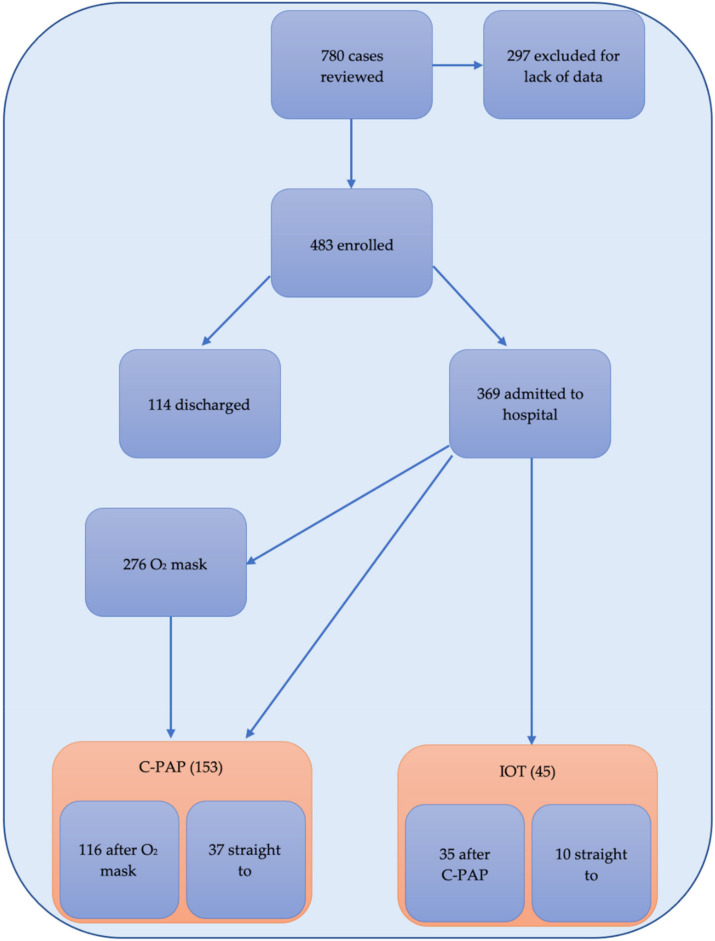
Flow chart of patients and oxygen strategies observed reporting enrolled patients; those discharged vs those admitted to hospital and the number of those admitted requiring oxygen mask, C-PAP and mechanical ventilation (IOT).

Between 1 March and 31 May 2020, 780 individuals were eligible for the study. Of these, 483 were enrolled for full analysis whilst 269 excluded for lack of data or the presence of exclusion criteria. A total of 369 (76.40%) were admitted to hospital: initially 276 were managed with oxygen mask, 68 with C-PAP helmet, and 10 were intubated. Of the 346 in oxygen mask, 116 needed C-PAP during admission having an overall of 153 treated with C-PAP. Of these, 35 needed IOT and ICU admission having an overall of 45 patients who needed mechanical ventilation. (Figure 1).

### 3.1. Characteristic of Patients

Median population age was 74 (IQR 61–83), and 278 (57.56%) were male. Coexisting disorders, home medications, vital parameters, laboratory test and arterial blood gas analysis at admission are reported in Table 1. Hypertension was the most common comorbidity (*n* = 205, 42.44%) followed by vascular artery disease (*n* = 86, 17.81%), hyper-cholesterolemia (*n* = 58, 12.01%), diabetes (*n* = 49, 10.14%) and solid cancer with active treatment (*n* = 50, 10.35%). Most of the patients were hemodynamically stable with median SatO2 95% (IQR 91–97) and median respiratory rate of 20, (IQR 18–25). Laboratory tests showed lymphopenia (0.9 10⁹ cells/L IQR 0.6–1.2), increased D-dimer and C-reactive protein.

Overall, 266/483 experienced an adverse outcome: intra-hospital mortality was 38.07% (185/483), *n* = 45 (9.26%) patients were admitted to ICU for mechanical ventilation, *n* = 70 (14.40%) met the criteria for C-PAP failure, and *n* = 40 (8.23%) had a hospital readmission within 30 days from discharge.

### 3.2. Outcome and Blood Gas Analysis

Blood gas analysis values showed at time 0 in the population with and without adverse outcome, respectively: a median of PaO2 70 mmHg (IQR 61–83) versus 64 mmHg (IQR 54–77) (*p* < 0.001); a median of PaO2/FiO2 ratio of 314 (IQR 266–371) versus 251 (IQR 165–310) (*p* < 0.001); a median of SatO2 95% (IQR 93–97) versus 95% (IQR 90–97) (*p* < 0.001).

For those who required hospital admission, we identified 217 patients as group A (those who were not on C-PAP) and 140 patients as group B (those on C-PAP).

In any group, patients with SatO2 < 94% experienced a significantly higher rate of adverse outcome at time 0, group A and group B on C-PAP (see Table 2).

About PaO2/FiO2 ratio, categorized into the chosen threshold of <100, 100–200, 200–300, >300, we found significant differences in these groups in terms of adverse outcome mainly between those with 0–300 versus > 300 (see Table 3).

Regarding PaO2 values, we found, at time 0 in group A and group B, that not only those with PaO2 < 60 but also those with PaO2 > 100 had a higher rate of adverse outcomes with respect to those with PaO2 60–100 (see Table 4).

#### Logistic Analysis Results

The ORs derived from logistic regression models for at least one adverse outcome, in-hospital mortality and IOT of SatO2 < 94% versus SatO2 ≥ 94%, PaO2/FiO2 with thresholds of 100–200–300 and PaO2 < 60 and > 100 versus PaO2 60–100 are reported in Table 5.

## 4. Discussion

This study describes SatO2, PaO2/FiO2 ratio and PaO2 in a cohort of patients affected by COVID-19 regardless of oxygenation strategies chosen out of an ICU setting. We found that SatO2 < 94%, PaO2/FiO2 ratio < 300 and PaO2 < 60 and > 100 were strongly associated with adverse outcomes.

SatO2 levels have been correlated to in-hospital mortality in many studies and are considered a relevant index in the management of acute respiratory failure from COVID-19 [6,28,29]. In this study, at time 0 SatO2 < 94% had 1.98 OR not only for in-hospital mortality but for the overall outcomes, more significantly the risk is doubled for those already on facial mask supplemental oxygen (Group A) and tenfold for those on C-PAP (Group B). Even if this is consistent with the pathophysiology of the disease [30], evidences are controversial: in a multicenter cohort study, Aliberti et al. found no significant difference in SatO2 levels between those who succeeded and failed helmet C-PAP [31] whilst other studies found worst outcomes for lower SatO2 levels [19,32].

PaO2/FiO2 ratio results are very much in line with those from previous studies about acute respiratory failure COVID-19 or non-COVID-19 related [33,34]. For those with values < 300, we found a three- to fourfold correlation with adverse outcomes at time 0, on facial oxygen mask and on C-PAP. Interestingly, the odds ratio did not vary much between the categories <100, <200, <300 except for <100 on C-PAP that showed much higher odds. This could be explained by the gravity of respiratory failure in patients without a chance of mechanical ventilation. In the work of Villar et al. [35], since then confirmed in many other studies mainly from ICU settings, PaO2/FiO2 is considered a predictor of adverse outcomes [36,37]. However, experimental studies reported a nonlinear relationship between PaO2 and FiO2 due to the degree of ventilation-perfusion ratio and pulmonary shunts [38,39]. Furthermore, even considering a fixed degree of shunts, PaO2/FiO2 fluctuates unpredictably for PaO2 values > 100 mmHg and varies with the mathematical-experimental model considered [40,41]. Thus, it seems plausible that in our study PaO2/FiO2 values < 300 identified patients at higher risk of adverse outcome, but it was not clinically relevant for values between 0 and 300.

About PaO2 values, we found a significant correlation with adverse outcome both for PaO2 < 60 mmHg and >100 mmHg at time 0, for those on facial mask oxygen and those on helmet C-PAP (see Table 4), having grouped this cohort together with “out of range” PaO2 values (<60 mmHg plus >100 mmHg versus 60–100 mmHg) for logistic regression analysis. Looking at the worst PaO2 detected, OR analysis showed a two- to threefold higher risk for overall adverse outcome and IOT, but about the best PaO2, results did not reach a statistical significance (see Table 5).

As reported in Table 1, we did not find significant differences of hemodynamic parameters and ABG values between time 0, groups A and B. Furthermore, mortality and hospital readmission rate were similar in groups A and B. This is consistent with the pathophysiology of COVID-19 disease that rarely causes severe respiratory failure and shock [1,2,3,4,5,6,7,8,9,10,11,12,13,14,15,16,17,18,19,20,21,22,23,24,25,26,27,28,29,30,31,32,33,34,35,36,37,38,39,40,41,42]. Moreover, it suggests that a proper oxygen and ventilation management prevent adverse outcomes [43].

Among physicians, whilst hypoxia is a well-established index of poor outcomes in ARDS, both COVID-19 and non-COVID-19 related [44,45], hyperoxia is less considered as being potentially harmful in clinical practice in acutely ill adults other than those with COPD or chronic pulmonary diseases [22,46,47]. Even in an ICU setting, de Graff et al. found poor clinical response in term of FiO2 adjustment in ventilated hyperoxic patients [48]. High arterial oxygen level, especially after a period of sustained hypoxia as happens in case of late hospital presentation, can have detrimental effects on multiple organs functioning due to a reperfusion injury and adaptation system derangement [49,50].

Summing up, we observed that altered SatO2 and PaO2 values are accurate predictors of adverse outcome irrespective of the oxygen strategies used. This mirrors physiological oxygen tissues delivery as both SatO2 and PaO2 represent arterial oxygen content [51,52]. Further studies are needed to evaluate whether Hb and cardiac output may have a significant role in ARDS COVID-19 related.

## 5. Limitations

This study had several limitations. First, we were not able to obtain all the data needed, so many patients had to be excluded from the analysis. Second, we analyzed arterial blood gas variables present on the patients’ electronic chart reporting the lowest values for PaO2/FiO2 and SatO2% and the lowest and highest values for PaO2. This is a simplification of the real pathophysiology whilst a continuous monitoring and analysis could have led to a more precise and consistent result. However, it is hard but necessary to find a way to summarize parameters variations, and it is difficult to obtain data in a non-ICU setting where continuous monitoring is not possible. A future prospective standardized study may address this with less bias.

Finally, we have to consider that at the time of the first wave, no specific recommendations were in place in our hospital to manage COVID-19 respiratory failure in terms of oxygen, PEEP and FiO2 administration, and furthermore, many non-intensivist physicians were involved so that clinical variability and experience could have led to a different outcome.

## 6. Conclusions

In this retrospective analysis, we found that SatO2 < 94%, PaO2/FiO2 < 300 and PaO2 < 60 and PaO2 > 100 correlate with a worst outcome. Hyperoxia should be avoided as it was found to be detrimental. Improving the PaO2/FiO2 ratio if already below 300 did not seem to predict a better outcome. Thus, it seems wiser to keep SatO2 and PaO2 in normal range values rather than improve the PaO2/FiO2 ratio. Even if these results are pretty much in line with general indications and clinical practice, these thresholds could give few but precise indications for physicians facing respiratory failure in COVID-19 patient in a non-ICU setting.

## Figures and Tables

**Table 1:** Characteristics of patients enrolled for the study.

	All Participants (*n* = 483)	GROUP A Admitted to Hospital Not Requiring C-PAP (*n* = 217)	GROUP B Admitted to Hospital on C-PAP (*n* = 140)
Age, years	74 (61–83)	77 (61–85)	69 (61–77)
Gender			
Men Woman	278/483 (57.56%) 205/483 (42.44%)	170/330 (51.52%) 160/333 (48.48%)	108/153 (70.59%) 45/153 (29.41%)
Coexisting Disorders			
Hypertension	205/483 (42.44%)	132/330 (40.00%)	73/153 (47.71%)
Smoke	17/483 (3.52%)	10/330 (3.03%)	7/153 (4.58%)
Hypercholesterolemia	58/483 (12.01%)	42/330 (12.73%)	16/153 (10.46%)
Heart failure with EF < 50%	18/483 (3.73%)	16/330 (4.85%)	2/153 (1.31%)
COPD	28/483 (5.80%)	21/330 (6.36%)	7/153 (4.58%)
Pulmonary restrictive diseases	2/483 (0.41%)	1/330 (0.30%)	1/153 (0.65%)
Coagulopathy	3/483 (0.62%)	2/330 (0.61%)	1/153 (0.65%)
Immunodepression (acquired or congenital)	22/483 (4.55%)	13/330 (3.94%)	9/153 (5.88%)
Diabetes	49/483 (10.14%)	26/330 (7.88%)	23/153 (15.03%)
Vascular artery diseases	86/483 (17.81%)	66/330 (20.00%)	20/153 (13.07%)
Chronic kidney diseases	22/483 (4.55%)	13/330 (3.94%)	9/153 (5.88%)
Medications			
ACE-inhibitors	104/483 (21.53%)	63/330 (19.09%)	41/153 (26.80%)
Steroids	25/483 (5.18%)	12/330 (3.64%)	13/153 (8.50%)
Active solid cancer	50/483 (10.35%)	37/330 (11.21%)	13/153 (8.50%)
Active hematological disorders	27/483 (5.59%)	18/330 (5.45%)	9/153 (5.88%)
Vital parameters at admission			
Systolic, mmHg	130 (117–145)	130 (116–145)	130 (120–150)
Diastolic, mmHg	75 (65–84)	75 (65–82)	77 (68–87)
SatO_2_, %	95 (91–97)	95 (92–97)	94 (89–97)
Heart rate, per minute	85 (75–99)	85 (75–99)	86 (75–98)
Respiratory rate, per minute	20 (18–25)	20 (18–24)	22 (18–30)
Temperature, Celsius	36.9 (36.5–37.7)	36.8 (36.5–37.7)	37 (36.5–37.7)
Laboratory test at admission			
White cell count, 10⁹ cells/L	6.98 (4.94–10.5)	7.18 (4.96–11.42)	6.77 (4.94–9.76)
Neutrophil, 10⁹ cells/L	5.3 (3.6–8.5)	5.4 (3.5–9)	5.25 (3.8–8.1)
Lymphocyte, 10⁹ cells/L	0.9 (0.6–1.2)	0.9 (0.6–1.3)	0.8 (0.5–1.05)
Hemoglobin, g/L	135.5 (122–147)	133 (119.5–144.5)	141 (125–150)
Platelets, 10⁹ cells/L	200 (153–266)	212 (156–275)	182 (139–243)
aPTT, second	33.1 (30.6–35.5)	32.7 (30.3–35.7)	33.45 (31.35–35.4)
INR	1.2 (1.12–1.32)	1.2 (1.11–1.34)	1.21 (1.14–1.29)
D-dimer, ng/mL	1027.5 (613.15–1636)	1054 (615.7–1969)	988 (612.6–1388)
Fibrinogen, g/L	5.47 (4.43–6.88)	5.2 (4.23–6.62)	6.26 (5.04–7.6)
C-reactive protein, µg/dL	75.4 (34.6–130)	64.7 (27.7–124)	99.15 (54.1–140)
Procalcitonin, ng/mL	0.15 (0.06–0.38)	0.12 (0.05–0.35)	0.175 (0.1–0.415)
Lactic dehydrogenase, u/L	307 (236–408)	289 (221–380)	339.5 (272.5–459.5)
IL-6, ng/L	638.85 (618.7–674.2)	632.2 (615.9–663.3)	655.8 (629.3–699)
Creatine-kinase, u/L	101.5 (59–204)	89 (54–160)	130 (68–238)
Ferritin, mg/mL	569 (264–1115)	497 (234–924)	758 (365–1425)
Troponin, µg/L	0.015 (0.015–0.043)	0.015 (0.015–0.066)	0.015 (0.015–0.026)
NT-proBNP, ng/L	350 (92–2076)	399 (91–2915)	302.5 (92–781)
Arterial blood gas analysis at admission			
pH	7.46 (7.42–7.49)	7.45 (7.42–7.49)	7.46 (7.43–7.49)
PaO_2_, mmHg	67 (58–80)	70 (61–83)	60 (52–73)
PaO_2_/FiO_2_ ratio	285 (203–340)	304.5 (232–357)	246 (150–294)
Outcomes			
Mechanical ventilation (IOT)	45/483 (9.26%)	10/330 (3.03%)	35/153 (22.88%)
Intra-hospital mortality	185/483 (38.07%)	123/330 (37.5274%)	60/153 (39.22%)
C-PAP failure	70/483 (14.40%)	0/330 (0%)	70/153 (45.75%)
Hospital readmission within 30 days from discharge	40/483 (8.23%)	25/330 (7.58%)	15/153 (9.80%)
Length of hospital stay, days	13.99 (3.98–23.14)	12.14 (1.95–22.05)	17.65 (11.14–24.04)

Table 1 reports all variables analyzed at time 0 of all patients, in GROUP A (those who were admitted to hospital non requiring C-PAP) and in GROUP B (those who were admitted to hospital on C-PAP. Data are reported as median with interquartile range (IQR) for continuous variable and with number and percentage (%) for non-continuous variables.

**Table 2:** Number of patients based on SatO2 values at time 0, in Group A and Group B, divided into those with and without at least one adverse outcome.

	Time 0		Group A		Group B	
At least one adverse outcome	No	Yes	No	Yes	No	Yes
SatO_2_ < 94%	59	113	18	75	1	34
SatO_2_ ≥ 94%	158	153	49	58	36	64
*p*-value	0.000		0.000		0.000	

**Table 3:** Number of patients based on PaO2/FiO2 values at time 0, in Group A and Group B, divided into those with and without at least one adverse outcome.

	Time 0		Group A		Group B	
At least one adverse outcome	No	Yes	No	Yes	No	Yes
PaO2/FiO2 < 100	7	30	12	60	2	41
PaO2/FiO2 100–200	20	59	30	68	23	44
PaO2/FiO2 200–300	65	96	17	14	6	14
PaO2/FiO2 > 300	125	81	10	6	6	4
*p*-value	0.000		0.000		0.000	

**Table 4:** Number of patients based on PaO2 values at time 0, in Group A and Group B, divided into those with and without at least one adverse outcome.

	Time 0		Group A				Group B			
	pO_2_		Best pO_2_		Worst pO_2_		Best pO_2_		Worst pO_2_	
At least one adverse outcome	No	Yes	No	Yes	No	Yes	No	Yes	No	Yes
pO_2_ < 60 mmHg	43	100	2	27	23	84	0	6	3	38
pO_2_ 60–100 mmHg	158	137	35	60	41	42	4	24	19	35
pO_2_ > 100 mmHg	16	29	29	47	3	8	32	68	14	25
*p*-value	0.000		0.005		0.000		0.055		0.003	

**Table 5:** OR values (95% CI) related to the comparison of SatO2 < 94% versus SatO2 ≥ 94%, of PaO2/FiO2 category with a threshold of 100, 200 and 300 and of PaO2 < 60 and > 100 versus PaO2 60–100 for the presence of at least one adverse outcome and in-hospital mortality.

	At Least One Adverse Outcome		In-Hospital Mortality	
	OR (95% CI)	*p*-Value	OR (95% CI)	*p*-Value
SatO2 < 94%				
at Time 0	1.98 (1.34–2.91)	0.001	2.12 (1.45–3.11)	0.000
in Group A	3.52 (1.86–6.67)	0.000	4.10 (2.25–7.47)	0.000
in Group B	19.12 (2.51–145.63)	0.004	10.12 (4.08–25.14)	0.000
PaO2/FiO2 at Time 0				
<100 vs. ≥100	3.81 (1.64–8.86)	0.002	3.33 (1.65–6.72)	0.001
<200 vs. ≥200	3.54 (2.19–5.70)	0.000	3.10 (2.02–4.77)	0.000
<300 vs. ≥300	3.10 (2.13–4.51)	0.000	3.40 (2.27–5.10)	0.000
PaO2/FiO2 in Group A				
<100 vs. ≥100	3.24 (1.60–6.55)	0.001	3.38 (1.88–6.09)	0.000
<200 vs. ≥200	4.11 (2.09–8.08)	0.000	2.47 (1.20–5.09)	0.014
<300 vs. ≥300	4.01 (1.39–11.54)	0.010	1.61 (0.54–4.81)	0.392
PaO2/FiO2 in Group B				
<100 vs. ≥100	11.57 (2.64–50.76)	0.001	11.94 (5.07–28.13)	0.000
<200 vs. ≥200	2.27 (0.96–5.33)	0.061	12.55 (2.85–55.28)	0.001
<300 vs. ≥300	4.79 (1.27–18.07)	0.021	**	
PaO_2_ < 60 or PaO2 > 100				
at Time 0	2.52 (1.72–3.70)	0.000	2.59 (1.77–3.79)	0.000
Worst in Group A	3.45 (1.87–6.37)	0.000	3.37 (1.81–6.26)	0.000
Best in Group A	1.39 (0.77–2.51)	0.272	1.13 (0.64–1.99)	0.671
Worst in Group B	2.01 (0.93–4.36)	0.077	3.72 (1.68–8.23)	0.001
Best in Group B	0.38 (0.12–1.20)	0.100	0.41 (0.17–0.95)	0.039

** Logistics find this as a perfect predictor for in-hospital mortality.

## Data Availability

The data presented in this study are openly available in our database (repository: Stefano Sartini, UOC MECAU, Ospedale Policlinico San Martino, Genova).

## Abbreviations

ARDS	acute respiratory distress syndrome
HFNC	high flow nasal cannula
C-PAP	continuous positive air pressure
NIV	non-invasive ventilation
ICU	Intensive Care Unit
DO2	tissue oxygen delivery
VO2	cellular oxygen consumption
ROS	reactive oxygen species
SatO2	Peripheral oxygen saturation
ABG	arterial blood gas
PaO2	partial pressure of arterial oxygen
PaO2/FiO2	partial pressure of arterial oxygen/fraction of inspired oxygen rate
RT-PCR	reverse transcription of polymerase chain reaction
COPD	Chronic obstructive pulmonary disease
ACE	angiotensin-converting enzyme
aPTT	activated partial thromboplastin time
INR	International Normalized Ratio
NT-proBNP	N-Terminal Fragment of the Prohormone Brain-Type Natriuretic Peptide
IOT	mechanical ventilation
IQR	interquartile range
OR	odds ratio
CIs	confidence intervals
